# Documentation of adverse events in non-commercial trials of intravitreal injection of anti-VEGF drugs to treat wet age-related macular degeneration (AMD)

**DOI:** 10.1186/1745-6215-12-S1-A13

**Published:** 2011-12-13

**Authors:** Barnaby C Reeves, Wendy Underwood, Heike Cappel-Porter, Sarah Baos, Chris A Rogers, Michael Arnott, Simon P Harding, Usha Chakravarthy, Alex E Foss

**Affiliations:** 1 Clinical Trials and Evaluation Unit, University of Bristol, Bristol, BS2 8HW, UK; 2 Department of Eye and Vision Sciences, University of Liverpool, Liverpool, L69 3BX, UK; 3 Centre for Vision and Vascular Sciences, Institute of Clinical Science, Queen’s University of Belfast, Belfast, BT12 6BA, UK; 4 Department of Ophthalmology, Nottingham University Hospitals, Queen’s Medical Centre, Nottingham, NG7 2UH, UK

## Objectives

IVAN and TANDEM are factorial randomised controlled trials (RCTs) of different treatment regimens for wet AMD, involving off-label use of bevacizumab. Safety data are being collected but, given follow-up visits every 4-8 weeks for up to 3 years and verbatim reporting, are difficult to collate. We have developed a database solution for the TANDEM trial to minimise duplicate adverse events or events without resolution dates, based on lessons learnt in the IVAN trial.

## Methods

Duplicate reporting of events can arise over multiple visits for several reasons, for example:

• different verbatim reporting (or typographic errors) of the same event;

• evolution of an event through stages of symptom, investigation, diagnosis, treatment;

• ongoing events may not be reviewed at every visit and resolution dates may be missed; if the data record for such an event is incomplete over time, it is impossible to know whether a subsequent report describes the original event or a recurrence.

## Results

The database solution has the following features:

• coded categories to match most common adverse events and mapped to MedDRA preferred terms, with an option to classify as ‘serious’, with reason;

• ‘other’ category;

• optional free-text qualification field for any event.

‘Other’ events will be reviewed regularly and additional categories added for common events not covered by the existing codes.

After the first trial visit, a customised adverse event data form is printed from the trial database in advance of interviewing the participant. The top half of the form requires information about ongoing events to be updated, allowing evolution of an event to be described. The bottom half of the form allows new events to be documented using the coded categories and qualification field. Failure to update an ongoing event generates an automatic data validation query.

## Conclusions

This solution provides complete transparency of adverse event reporting and real-time coding. It requires ongoing events to be updated and minimises the problems created by free-text reporting.

